# Can Body Condition and Somatic Indices be Used to Evaluate Metal-Induced Stress in Wild Small Mammals?

**DOI:** 10.1371/journal.pone.0066399

**Published:** 2013-06-18

**Authors:** Nicolas Tête, Clémentine Fritsch, Eve Afonso, Michaël Coeurdassier, Jean-Claude Lambert, Patrick Giraudoux, Renaud Scheifler

**Affiliations:** 1 Department of Chrono-Environment, UMR UFC/CNRS 6249 USC INRA, University of Franche-Comté, Besançon, France; 2 Institut Universitaire de France, Paris, France; University of Western Ontario, Canada

## Abstract

Wildlife is exposed to natural (e.g., food availability and quality, parasitism) and anthropogenic stressors (e.g., habitat fragmentation, toxicants). Individual variables (e.g., age, gender) affect behaviour and physiology of animals. Together, these parameters can create both great inter-individual variations in health indicators and interpretation difficulties. We investigated the relevance of body condition and somatic indices (liver, kidneys) as indicators of health status in wood mice (*Apodemus sylvaticus*, *n = *560) captured along a metal pollution gradient in four landscape types (30 sampling squares 500-m sided). The indices were calculated using a recently proposed standard major axis regression instead of an ordinary least square regression. After considering age and gender for the body condition index, no landscape type influence was detected in the indices. However, important index variability was observed between sampling squares; this effect was included as a random effect in linear models. After integrating all individual and environmental variables that may affect the indices, cadmium (Cd) concentrations in both the liver and kidneys were negatively related to body condition and liver indices only for individuals from highly contaminated sites. Lead in the liver was negatively related to the liver index, and Cd in kidneys was positively linked to the kidney index, potentially suggesting metal-induced stress. However, interpretation of these indices as a wildlife ecotoxicology tool should be performed with caution due to the sensitivity of potentially confounding variables (e.g., individual factors and environmental parameters).

## Introduction

A wide range of morphological, biochemical, and physiological metrics have been developed as health indices [1]. Body condition is commonly defined as a measure of the energetic or nutritional state of an animal [2], [3]. Calculations and interpretation of body condition indices (BCI), which are often based on the relationship between body mass and length measurements, are highly debated, and BCI have been found to correlate with fitness parameters related to reproduction and survival in mammals and other taxa [4]. Individual quality, defined as an estimate of individual fitness, has been estimated by using somatic indices, i.e., the relative size of internal organs [5]. For instance, liver size and pancreas size have been shown to correspond to low quality foraging in herbivorous mammals [5].

Among the numerous markers developed to assess deleterious effects of trace metals (TMs) in wild small mammals, body condition and somatic indices have sometimes been used, with inconsistent results. For instance, Sanchez-Chardi et al. [6] observed that the body condition index and the relative liver weight (i.e., tissue weight divided by body weight) tended to decrease in adult wood mice, *Apodemus sylvaticus*, from a landfill site, while the relative kidney weight increased [6]. In the same site, no difference of morphological parameters (both body condition and somatic indices) was found in the greater white-toothed shrew, *Crocidura russula* [7]. Nunes et al. [8] found that body length was higher in *Mus spretus* mice from a reference site compared to mice inhabiting a metal-contaminated area. Body weight and BCI were influenced by both gender and reproductive activity, but not by the level of pollution. However, kidney and spleen relative weights were larger in the reference site [8].

These various studies may have suffered from several limitations that potentially explain these discrepancies. First, the sample sizes were relatively low (52< *n* individuals <147) considering that multiple variables (gender, age, site, etc.) may impact BCI and somatic indices. Moreover, the calculations of BCI and somatic indices did not consider the variation in the relationship between mass and length as body size changes and growth occurs (allometry), which was recently noted as a potentially misleading bias [9], [10]. In these studies [9], [10], the authors proposed a new index calculation using a standard major axis (SMA) regression instead of the commonly used ordinary least square regression. Scaled mass index (SMI, defined as body condition index calculated following the SMA procedure) was shown to be a better indicator of the relative size of energy reserves and other body components compared with ordinary least square residuals [9], [10]. Finally, in the comparison of individuals from only one contaminated *versus* one reference area, site characteristics and confounding variables such as variations in resources availability and habitat or landscape features might influence the relationship between BCI/somatic indices and contamination, even though most contaminated and control study sites were selected for similar vegetation and climate conditions.

In this context, the aim of this work was to study the relationships between scaled mass index and somatic indices and concentrations of TMs in the tissues of wood mice from 30 sampling sites surrounding a large-scale smelter-impacted area in Metaleurop Nord, northern France. A large sampling effort was needed to simultaneously study the influence of several variables in a sufficient sample size. These variables include individual characteristics (age and gender) as well as metal concentrations in the soil and in two organs: the liver and kidneys. Landscape type has been shown to influence small mammal population dynamics and community composition, structure and dynamics [11]–[15]; thus, this variable was included in the models to test the potential modulation of body condition or somatic indices.

## Materials and Methods

### Study Site

**Figure 1:**
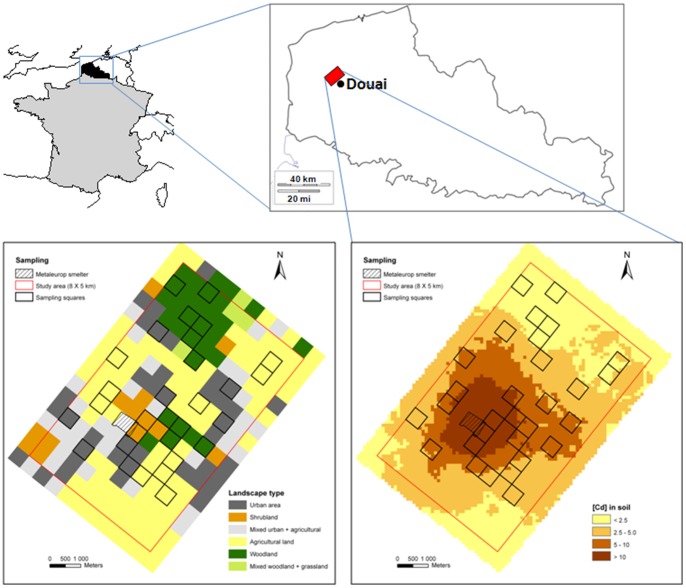
Study site localisation, Cd contamination (µg/g DM) in soils and landscape types. Study site located around the former Metaleurop Nord smelter (Nord-Pas de Calais, France); maps present the landscape types in each square and the Cd concentrations in the study area soils. Selected squares for wood mice sampling are bolded in both maps.

**Figure 2:**
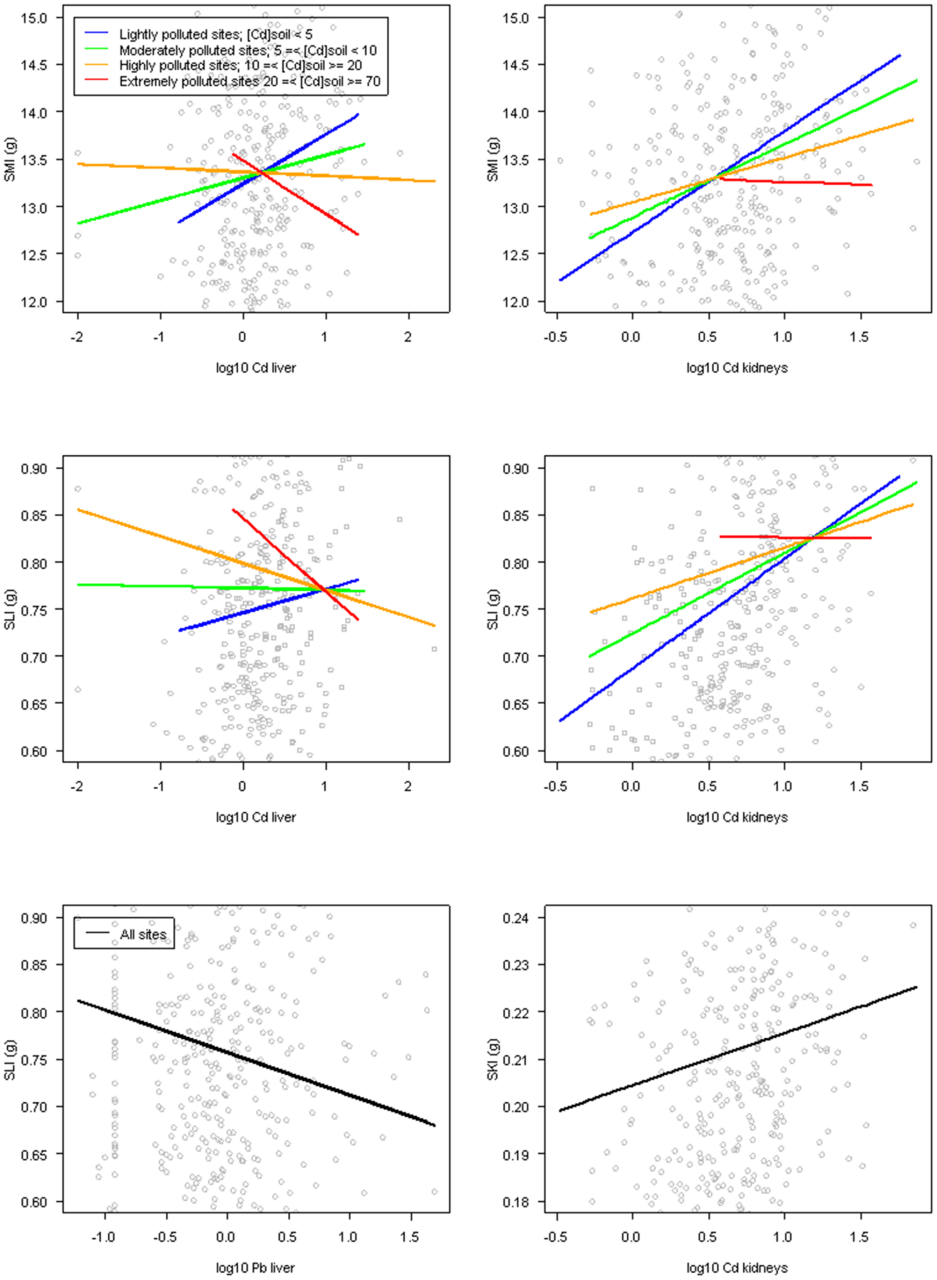
Predicted relationships between indices and metal concentrations in organs (µg/g DM). Predictions between indices and TM concentrations are performed on wood mice from the former Metaleurop Nord smelter; (a) between SMI and [Cd]_liver_; (b) between SMI and [Cd]_kidneys_; (c) between SLI and [Cd]_liver_; (d) between SLI and [Cd]_kidneys_; (e) between SLI and [Pb]_liver_; and (f) between SKI and [Cd]_kidneys_. For all figures, the age of individuals was fixed (7 mg of crystalline lens mass, which corresponds to adulthood). For graphical representation, the “highly polluted” class was subdivided into two classes of contamination, “highly polluted” (10≤ [Cd]_soil_ ≥20 µg/g DM) and “extremely polluted” (20≤ [Cd]_soil_ ≥70 µg/g DM) in figures (a) to (d). For figures (a) and (b), predicted relationships are shown only for males. For figures (a) to (d), relationships between indices and TM concentrations are represented at different levels of soil contamination (lightly, moderately, highly and extremely polluted). Because interactions between concentrations in organs and soil were not significant in figures (e) and (f), predicted relationships are drawn for all contaminations levels.

Fieldwork was conducted around the former lead (Pb) and zinc (Zn) Metaleurop Nord smelter (Noyelles-Godault, Nord-Pas-de-Calais, northern France, 50°25′42 N 3°00′55 E). The study area, spanning 40 km^2^ (Figure 1) around the former smelter, was divided into 160 squares of 500×500 m that constituted our sampling units. Smelting activity for more than 100 years has caused dramatic pollution in this area by three main TMs: cadmium (Cd), Pb, and Zn [11], [16]. To evaluate the concentration of TMs in the soil (0–25 cm depth), a composite soil sample (15 points in homogeneous woody patch) was taken in one to ten woody patches (for instance, hedgerows, tree plantations, copses, or woodlots) in each square during the autumn of 2006. Soil metal levels in the soil site ranged from 0.1 to 2,402 µg/g of dry matter (DM) for Cd, from 16 to 41,960 µg/g DM for Pb, and from 44 to 38,760 µg/g DM for Zn [11]. Three levels of soil contamination, defined as “light” (median [Cd] ≤5 µg/g DM soil and median [Pb] ≤300 µg/g DM soil), “moderate” (5< median [Cd] ≤10 µg/g DM soil and/or 300< median [Pb] ≤600 µg/g DM soil), and “high” (median [Cd] >10 µg/g DM soil or median [Pb] >600 DM µg/g soil), were allocated to each square. For graphical representation (Figure 2a to d), the “highly polluted” class was subdivided into two classes of contamination, “highly polluted” (10≤ [Cd]_soil_ ≥20 µg/g DM soil) and “extremely polluted” (20≤ [Cd]_soil_ ≥70 µg/g DM soil).

A land use analysis was performed to determine the landscape composition of each square [17]. According to their dominant land cover, seven types of landscapes were identified throughout the area: urban area, shrubland, mixed urban/agricultural land, agricultural land, woodland, mixed woodland/grassland, and the Metaleurop former smelter (Figure 1). Only the four landscape types mainly represented in the area (dominated by agricultural land, urban areas, woodland, and shrubland) were studied here.

### Small Mammals Sampling

During the autumn of 2006, small mammals were trapped in 30 squares selected from among the 160 squares of the grid to obtain several sampling square replicates for each pollution level in each landscape type (Figure 1, Table 1). Sampling was performed in the woody patches, where soil concentrations have been previously measured. In each sampling square, 10 lines of 10 3 m-spaced break-back traps were set. In 3 squares, the available areas of woody patches were insufficient to place 10 trap-lines; thus, the number of lines was reduced to 6–7 in these squares. The 290 trap-lines were checked every morning for three consecutive days and re-set/re-baited if needed. The sampling effort consisted of 2900, 580, 2610, and 2610 trap-nights in the ‘agricultural land’, ‘shrubland’, ‘urban’, and ‘woodland’ landscape types (surrounding the woody patches where animals were trapped), respectively. The percentage of captures, hereafter referred to as “capture success”, was calculated as the number of individuals trapped per 100 trap-nights. Sampling authorisation was given by the *Direction Régionale de l’Environnement, de l’Aménagement et du Logement* (DREAL) of Nord – Pas-de-Calais. Captured animals were frozen following capture and stored at −20°C until dissection.

**Table 1:** Pollution level, median Cd and Pb soil concentrations (µg/g DM), and the number of sampling squares for each landscape type studied.

		Landscape type										
		Agricultural lands			Shrublands		Urban areas			Woodlands		
Pollution level	+	++	+++	+	++	+++	+	++	+++	+	++	+++
Number of sampling squares	3	4	5	0	0	2	1	2	3	6	2	2
Median [Cd]_soil_ (µg/g)	3.7	5.3	13.8			54.1	3.9	4.8	16.3	1.5	8.3	10.1
Median [Pb]_soil_ (µg/g)	189	337	710			1851	259	313	1392	179	548	416

### Animal Preparation and Morphometric Measurements

Animals were thawed, identified to specific levels based on morphometrics and skull and teeth characteristics [18], [19], weighed, measured, dissected, sexed, and aged. The wood mouse, *Apodemus sylvaticus*, whose biomass largely dominates the community [20], is the only species studied in this work. Liver and kidneys were weighed. The age of rodents was estimated by measuring their crystalline dry weight (following Quéré and Vincent [21]).

Because body length measurement was problematical (heads were cut off prior to dissection), the left foot length was measured. Measurements were performed from the heel to the central claw, to the nearest 0.01 mm. However, the body length of small animals is considered to be a more suitable measure of structural length than foot length and is preferred for SMI computation [9]. The body length was predicted from the foot length using a linear model based on wood mice captured during another sampling session (autumn 2010) conducted in the same study site. Body and foot lengths were measured in 192 individuals following the exclusion of females showing reproductive traits and of individuals partially eaten in the traps. Body length was predicted using a linear model including “foot length”, “age”, “gender”, and interactions between foot length, age, foot length, and gender as independent variables (Pearson r^2^ = 0.83, *p*<0.001). Body lengths of individuals captured in 2006, referred to as “body length” hereafter, were predicted from their foot lengths with the equation of the model described above.

The entire body (body and head) was weighed (to the nearest 0.01 g); after dissection, the digestive tract was subtracted prior to the calculation of the indices. Females showing reproductive traits (pregnancy or lactation) were excluded from the dataset to avoid biases in weight estimation due to physiology.

### Measure of TM Concentrations in Tissues

Metal concentrations (Cd, Pb) were measured in the liver and kidneys by furnace atomic absorption spectrometry (AAS, VARIAN 240Z) and expressed as µg/g dry mass (DM). Samples were digested with nitric acid (5 mL HNO_3_, 65%, Carlo-Erba, analytical quality) in a drying oven (65°C) during 72 h. After digestion, samples were diluted by adding 20 mL ultra-pure water (Elga, 18.2 MΩ/cm^2^). Blanks (acid+water) and certified reference materials (CRM: TORT-2, lobster hepatopancreas, and DOLT-3, dogfish liver, from the National Research Council Canada) were prepared and analysed with the samples. Duplicates were performed for each analysis and repeated if the RSD was above 5%. Average CRM recoveries were calculated at 98±28% for Pb and 100±17% for Cd. Lead and Cd detection limits in organs were 0.24 µg/g DM and 0.01 µg/g DM for the liver, and 1.03 µg/g DM and 0.04 µg/g DM for kidneys, respectively. When a concentration value was under the detection limit, half of the detection limit value was used for statistical analyses.

The percentage of individuals considered at risk for metal-induced stress was estimated according to the thresholds proposed by Shore and Douben [22], [23] as follows: number of individuals with Cd or Pb internal concentrations>thresholds×100)/total number of individuals (Table 2).

**Table 2:** For each soil contamination level, concentrations of Cd and Pb (minimum, mean, and maximum values, µg/g DM) in the liver and kidneys and number and percentage (in brackets) of wood mice at risk for metal-induced stress from the surroundings of the former Metaleurop Nord smelter.

Organs	Pollution level	*n*	Cd (µg/g DM)			Pb (µg/g DM)			Cd thresholds (µg/g DM)	Pb thresholds(µg/g DM)	Number (percentage) of individuals at risk for metal-induced stress	
			Min	Mean	Max	Min	Mean	Max			Cd	Pb
Kidneys	+	121	0.33	5.4	68	0.52	3.4	26	105	25	0	1 (0.8%)
	++	178	0.35	5.5	154	0.52	8.1	151			1 (0.6%)	16 (9.0%)
	+++	261	0.53	8.8	332	0.52	43.2	1282			4 (1.5%)	63 (24%)
Liver	+	122	0.14	2.5	38	0.12	0.72	13	15–140	10	4 (3.3%)–0	1 (0.8%)
	++	178	0.01	4.1	209	0.12	0.95	14			12 (6.7%)–1 (0.6%)	2 (1.1%)
	+++	260	0.01	3.7	91	0.12	3.4	50			24 (9.2%)–0	21 (8.1%)

Individuals are considered at risk for metal-induced stress when TM concentrations were above the thresholds defined by Shore and Douben [22], [23].

### Indices Calculations and Statistical Analyses

Potential differences in population structure (sex ratio and mean age differences as estimated by crystalline lens mass) between pollution levels and landscapes were checked using χ^2^ tests for the sex ratio and a Fisher test for age.

Relationships between TM concentrations (log10-transformed) in soils and organs were studied using a Pearson correlation test after graphically checking the normality of residuals of linear models and the variance homoscedasticity.

Body condition and somatic indices were calculated for 653 individuals out of the 859 individuals sampled after the suppression of partially eaten individuals and pregnant or lactating females [3]. Body condition was estimated using the method proposed by Peig and Green [9], [10] based on a standardised regression axis (SMA) instead of an ordinary least squares (OLS) regression between individuals. The SMI standardises body mass at a fixed value of a linear body measurement based on the scaling relationship between mass and length according to the equation *SMI* = *m_i_*(*L*_0_/*L_i_*)*^b^* ^SMA^, where *m_i_* and *L_i_* are the body mass and the linear body measurement of an individual *i*, respectively; *b*_SMA_ is the scaling exponent estimated by the SMA regression of ln*M* on ln*L*; *L*_0_ is an arbitrary value of *L* (e.g., arithmetic mean value for the study population); and *SMI* is the predicted body mass for individual *i* when the linear body measure is standardised to *L_0_* [9], [10]. For the purpose of homogeneity, somatic indices were built using an SMA regression. Somatic indices will hereafter be referred to as the scaled liver index (SLI, for the somatic index of the liver) and the scaled kidney index (SKI, for the somatic index of kidneys). In this study, the values of *b*_SMA_ were 3.14, 1.40, and 0.45 for SMI, SLI, and SKI, respectively. The *b*_SMA_ value for SMI slightly varied among sites: 3.50 for individuals from weakly polluted sites, 2.99 for moderately polluted sites and 3.06 for highly polluted sites; however, those differences were not significant (*p*>0.050). Although Peig and Green [10] suggested using only data from uncontaminated sites to estimate the *b*_SMA_ for the scaled mass index, the entire population *b*_SMA_ value was used in the present study because *b*_SMA_ did not differ among sites. In the literature, the *b*_SMA_ value for SMI lies within the range of 2.5–3.2, which is estimated to be a guideline to identify reliable estimates of the allometric exponent in mammals [9], [10]. The same authors found a value of 2.71 (95% confidence interval: 2.43–2.99) for 97 wood mice individuals captured in an unpolluted site in northeastern Spain. The estimated *b*_SMA_ in both organs was remarkably constant along the pollution gradient (from 1.31 to 1.40 for SLI, and from 0.44 to 0.48 for the SKI). No comparable value of ontogenetic allometric exponents for the liver and kidneys was found in the literature. Correlations between SMI, SLI, and SKI were studied using a Pearson correlation test.

Linear mixed models were used to analyse the relationship between SMI or somatic indices and predictor variables. The spatial dependence of body condition and somatic indices was studied by computing omnidirectional empirical variograms with variographic envelops obtained by permutation (99 simulations, Monte-Carlo method [24]). None of the variograms showed a spatial structure, and no significant spatial auto-correlation was detected. Therefore, spatial correlation structures were not included in further models. Individuals from the same sampling square were assumed to be exposed to the same environmental constraints acting on indices, and the indices were highly variable between sampling squares; therefore, a variable “sampling square” was included as a random effect in the models. Even though the SMA regression used to calculate the indices is supposed to consider allometry, age and gender were still included in the models. Age, gender, landscape, and TM concentrations in organs and soil were included in the models as fixed predictor variables using the following procedure: age and gender were first considered in the models as single and second order interaction terms. The effect of age on indices was tested as a linear relationship to indices throughout life and as a polynomial function of degree 2 (increasing until adulthood and then decreasing for oldest ages). Finally, TM concentrations in organs, as well as their second order interactions with age, gender, landscape, and TM concentrations in soil, were included in the models. Random and fixed effects were tested with a likelihood ratio test (LRT, *p*<0.050). Fixed effects residuals of the final models were graphically checked for normality and variance homoscedasticity. The likelihood ratio R^2^ [25] was calculated to determine which part of the index variability was due to fixed effects. The proportion of random effects variance (hereafter referred to as V^2^) explained by the sampling square was estimated to evaluate the individual index variability between sampling squares.

All statistical analyses were performed with R 2.15.1 software with the additional libraries “lmmfit”, “lmodel2”, “nlme”, and “pgirmess” [26].

## Results

### Sample Size and Wood Mice Population Structure

During the field session, 859 wood mice were trapped. Although the global population was slightly dominated by males (54%), no difference in sex ratios was observed in different landscape types (χ^2^ = 4.5, ddl = 3, *p* = 0.210) or contamination levels (χ^2^ = 0.25, ddl = 2, *p* = 0.882). The capture success was 9.4% (*n* = 274), 11.7% (*n = *68), 5.6% (*n* = 147) and 14.2% (*n* = 370) in the landscape types of agricultural land, shrubland, urban area, and woodland, respectively. Capture success differed between landscape types (χ^2^ = 109.9, ddl = 3, *p*<0.001); fewer wood mice were captured in urban landscapes than in woodland landscapes. Capture success differed along the soil pollution gradient; success was higher in squares considered to be highly polluted than in moderately and lightly polluted squares (χ^2^ = 55.1, ddl = 2, *p*<0.001). Mean age differences (estimated by crystalline lens mass) were observed along the contamination gradient (*p* = 0.009); individuals from the moderately polluted site had a lower average age than in the other sites. Mean age differed between the four landscape types (*p*<0.001). On average, captured individuals were younger in agricultural and urban areas than in the two other landscape types.

### Accumulation of TMs in Wood Mice

Due to technical and time constraints, 560 individuals out of the 859 were randomly chosen for TM concentration measurements in organs. The concentrations of Cd and Pb measured in the liver and kidneys showed a high variability along the pollution gradient; the values ranged from 0.01 to 332 µg/g DM for Cd and from 0.06 to 1,282 µg/g DM for Pb (Table 2). Cadmium and Pb concentrations were positively correlated between the liver and kidneys (respectively, r = 0.77 for Cd, and r = 0.78 for Pb, *p*<0.001). Average Cd and Pb concentrations were 2.4 and 12 times higher, respectively, in kidneys than in the liver. Organ concentrations of Cd and Pb increased with increasing TM soil concentrations (r_Cdkidneys_ = 0.24, *p*<0.001; r_Cdliver_ = 0.14, *p* = 0.001; r_Pbkidneys_ = 0.53, *p*<0.001; r_Pbliver_ = 0.50, *p*<0.001). The percentage of individuals at risk for metal-induced stress increased along the contamination gradient (Table 2) and ranged from 0 to 3.3% in lightly polluted squares, 0.6 to 9% in moderately polluted squares, and 1.5 to 24% in most polluted squares. The percentage of individuals at risk for metal-induced adverse effects was largely influenced by high Pb concentrations in kidneys.

### Influence of Individual and Environmental Factors on Body Condition and Somatic Indices

The SMI varied negatively with the age of wood mice (0.15< R^2^_age_ <0.17, *p*<0.001, Table 3) and did not have a better fit with a polynomial relationship than with a linear one (LRT, *p* = 0.063). SMI for males were slightly higher than for females (mean SMI adjusted on age: females = −0.49, males = 0.06; *p = *0.035). The SMI decrease with age was sharper for females than for males (*p*<0.001). Landscape type did not influence the SMI (*p* = 0.066). After considering age, gender, and their second order interaction, the interaction between TMs in soil and TMs in organs was included in the models. The relationship between SMI and hepatic Cd concentrations varied depending on soil pollution, and a significant second order interaction existed between Cd concentrations in the liver and soil (R^2^_[Cdsoil]:[Cdliver]_ = 0.05, *p*<0.050). SMI increased with Cd concentrations in the liver for animals sampled in lightly and moderately polluted sites ([Cd]_soil_ ≤10 µg/g DM) and decreased for the most polluted sites (mean [Cd]_soil_ >10 µg/g DM; Figure 2a, Table 3). The same pattern was observed for the relationship between SMI and renal Cd concentration (R^2^_[Cdsoil]:[Cdkidneys]_ = 0.06, *p*<0.050; Figure 2b, Table 3); the SMI of specimens from extremely contaminated sites decreased. Organ Pb concentrations were not related to SMI or their interaction with Pb soil concentrations. In all selected models, the proportion of random effects variance explained by the sampling square was large (0.36 to 0.41), showing a high individual SMI variability between sampling squares.

**Table 3:** Model outputs presenting partial R_LR_^2^ and *p*-values in brackets (F-statistics) for each variable and for the entire model.

	TMsvariable	Age	Gender	[TM]_soil_	[TM]_organ_	Age:gender	[TM]_soil_:[TM]_organ_	R_LR_^2^(fixedeffects)	V^2^(randomeffects)
SMI	Cd liver	0.15 (*p*<0.001)	0.01 (*p* = 0.030)	0.02 (*p* = 0.028)	0.00 (*p* = 0.197)	0.05 (*p*<0.001)	0.03 (p = 0.005)	0.26	0.38
	Cd kidneys	0.15 (*p*<0.001)	0.01 (*p* = 0.027)	0.02 (*p* = 0.005)	0.02 (*p* = 0.004)	0.05 (*p*<0.001)	0.02 (*p* = 0.011)	0.28	0.36
	Pb liver	0.17 (*p*<0.001)	0.02 (*p* = 0.035)	NS	NS	0.06 (*p*<0.001)	NS	0.25	0.41
	Pb kidneys	0.17 (*p*<0.001)	0.02 (*p* = 0.035)	NS	NS	0.06 (*p*<0.001)	NS	0.25	0.41
SLI	Cd liver	0.05 (*p* = 0.001)	NS	0.00 (*p* = 0.170)	0.00 (*p* = 0.989)	NS	0.04 (*p* = 0.004)	0.11	0.32
	Cd kidneys	0.04 (*p* = 0.001)	NS	0.00 (*p* = 0.563)	0.07 (*p*<0.001)	NS	0.03 (*p* = 0.004)	0.14	0.30
	Pb liver	0.07 (*p* = 0.002)	NS	NS	0.05 (*p* = 0.008)	NS	NS	0.13	0.37
	Pb kidneys	0.13 (*p* = 0.002)	NS	NS	NS	NS	NS	0.13	0.35
SKI	Cd liver	0.27 (*p*<0.001)	NS	NS	NS	NS	NS	0.27	0.34
	Cd kidneys	0.25 (*p*<0.001)	NS	NS	0.01 (*p* = 0.023)	NS	NS	0.26	0.33
	Pb liver	0.27 (*p*<0.001)	NS	NS	NS	NS	NS	0.27	0.34
	Pb kidneys	0.27 (*p*<0.001)	NS	NS	NS	NS	NS	0.27	0.34

Models were constructed as follows: Index ∼ age+gender+[TM]soil+landscape+[TM]organ+age:gender+age:[TM]organ+gender:[TM]organ+[TM]soil:[TM]organ+landscape:[TM]organ. Because landscape and two-way interactions between [TMs] in organs and age or gender were not significantly related to the studied indices in all tested models (LRT, p>0.050), these variables were not included in the table. When a two-way interaction was significant, all terms (even not significant ones) included in this interaction are presented in the table.

The SLI was positively related to age (0.04< R^2^_age_ <0.13, 0.001<*p*<0.002), but no gender-related differences were detected. The landscape did not significantly influence SLI. After considering the effect of age, the same pattern described above for SMI was observed for the SLI. SLI and interactions between Cd liver and Cd soil and between Cd kidneys and Cd soil were significant (respectively, R^2^_[Cdsoil]:[Cdliver]_ = 0.04, *p* = 0.004 and R^2^_[Cdsoil]:[Cdkidneys = _0.10, *p*<0.010; Figure 2c and 2d, Table 3). SLI decreased with Pb concentrations in the liver regardless of Pb concentrations in soil (Figure 2e, Table 3). Finally, neither Pb concentration in kidneys nor its interaction with Pb concentration in the soil was related to SLI.

The SKI was positively influenced by age (0.25< R^2^_age_ <0.27, *p*<0.001), indicating that older wood mice had heavier relative kidney mass than young ones. None of the tested predictor variables and second order interactions were related to SKI for Cd concentrations in the liver or Pb concentrations in the liver and kidneys. However, SKI increased with Cd concentrations in kidneys (Figure 2f, Table 3).

Landscape types and second order interactions between TM concentrations in organs and age or gender were not related to the studied indices.

SMI, SLI, and SKI all positively correlated with each other (r_SMI/SLI_ = 0.30, *p*<0.001; r_SMI/SKI_ = 0.30, *p*<0.001; r_SLI/SKI_ = 0.61, *p*<0.001).

## Discussion

### Influence of Individual Variables on Body Condition and Somatic Indices: Methodological Considerations

Body condition and somatic indices are a much debated issue in ecology [2], [4], [9], [10]. Calculation methods and interpretations of these indices are subjects of numerous discussions, including those on conceptual (relationships between indices and fitness) and applied (for instance, the use of BCI as useful non-lethal tools for wildlife and conservation studies) issues. In the ecotoxicology of wild small mammals, such indices have been scarcely used and have given inconsistent, if not contradictory, results. In the present work, we calculated the indices using the SMA method that has been shown to represent a better indicator of the relative size of energy reserves and other body components than the ordinary least square residuals, which was the most widely accepted calculation of condition indices until recently [9], [10]. To enable meaningful comparison between individuals, this method of calculation of condition indices should remove the effects of ontogenetic growth (age) on the relationship between size and mass through standardisation [10]. Similarly, if males and females of a species exhibit a similar body shape, then differences in body condition indices between genders should not occur, even in the case of size dimorphism [10]. Contrary to the expected result of this new calculation, all the indices were influenced by age, and SMI was additionally influenced by gender. If the relationships of indices to age or gender are not due to experimental inaccuracies (e.g., measurements errors or prediction of body length from foot length), then the use of SMA models to consider allometric effects is insufficient to fully consider age- and gender-related patterns of mass/length relationships. It is reasonable to keep age and gender variables in models in an attempt to relate the indices, even “scaled mass” ones, to environmental variables of interest such as landscape and TM concentrations in the liver, kidneys and soil in the present case.

### Influence of Environmental Characteristics on the Indices

Our results showed that landscape type did not have a significant influence on SMI and somatic indices. To our knowledge, data considering the effect of landscape and/or habitat on body condition and somatic indices are scarce. The only relevant study we found is Tattersall et al. [27]: after cessation of breeding, male wood mice trapped in two sampling sites with the same characteristics had significant differences in body weight. The wood mice were significantly heavier in woodlands than along crop boundaries. In *Peromyscus leucopus*, an American species ecologically equivalent to *A. sylvaticus*, body condition (assessed as the proportion of body mass to body size) was influenced by habitat succession induced by the use of herbicide and/or burning, with better conditions in artificially induced early successional habitats [28]. The authors suggest that an overall improvement in the nutritional quality of habitats might be involved rather than an increase of quantity of food, and the authors notice that an interaction with season often influences the effect of habitat on body condition and body mass. To our knowledge, SLI and SKI variations have not been studied in relation to landscape. Some studies, however, have used food quality or multiannual population cycle phases in the wild to investigate the indirect effects of habitat on somatic indices. For instance, Klemola et al. [29] tested the hypothesis of Seldal et al. [30], which states that chemical (proteinase inhibitors) plant defence induced by intensive grazing may cause cyclic fluctuations in the densities of small mammals and other herbivores. For this purpose, these authors studied the size of internal organs (pancreas and liver) as an indicator of nutritional state. These authors, however, did not observe obvious differences in the relative size of organs related to cycle phase [29]. Nonetheless, laboratory studies have found that the quality of food might lead to organ hypertrophy or hypotrophy. Harju and Tahvanainen [31] showed that when adult field voles, *Microtus agrestis*, were fed for two weeks with diets with increased levels of the birch *Betula pendula,* a diet item consumed by voles in times of high population densities, the field voles exhibited increased liver size. In their study on *P. leucopus*, McMurry et al. [28] found heavier livers and spleens in adult males from artificially induced early successional habitats. Taken together, these results suggest that habitat, most likely through food quality, may influence both body condition and somatic indices in wild small mammals.

Our results additionally showed that indices were largely variable between sites, which may be related to local unknown environmental features and ecological factors. Inclusion of the random effect of model sampling squares allowed consideration of site-specific index variability. This result raises questions regarding the number of sampling sites required and the number of animals that should be trapped in each site to consider site-specific variability of the markers under study. In fact, comparison of individuals from only one contaminated site *versus* one reference site (as commonly observed in the literature), could lead to misinterpretation of results even if the sites are selected for their apparent similarities.

### Use of Somatic and Condition Indices as Contamination and Health Status Indicator

As the maximal internal Cd and Pb concentrations observed in this study are among the highest reported in wild rodents [6], [32]–[34], one could argue that deleterious effects, and thus responses of the indices, would be obvious in such a contaminated site. This work considered individual (age, and gender for SMI models) variables and found slight but significant relationships between both condition and somatic indices and contamination variables (Cd and Pb) in the liver, in the kidneys, and in the environment. The relationships between SMI and Cd concentrations in the liver and kidneys differed depending on the level of soil contamination. SMI increased with Cd concentrations in both the liver and kidneys of animals living in lightly and moderately polluted areas, suggesting that these animals did not exhibit any adverse effects of internal contamination. However, the relationship became negative for animals trapped in highly polluted sites ([Cd] ≥10 µg/g DM soil). Previous studies have shown diminution or reduction tendency in body weight, carcass weight, and/or body condition in individuals from small mammal species that inhabit polluted sites [6], [8], [34], [35]. Interpreted with other studied parameters, the decrease of this index in highly exposed animals was interpreted as a sign of poor health compared to the individuals living in a less polluted site. However, the attribution of these effects to TMs, other pollutants, or a combination of both remained speculative. The authors of several other studies failed to detect any significant differences in body condition/body weight in small mammals from polluted and reference sites [36], [37]. The present results reveal complex relationships of SMI with both individual (age, gender, TM concentrations in tissues) and environmental (high intra- and inter-site variability) variables. Confounding variables included in this study contribute to an explanation of the discrepancies observed in the literature.

As for the SMI, results showed a slight but significant relationship between SLI and the Cd concentrations in kidneys; this relationship was dependent on the level of soil contamination. SLI increased with Cd in both the liver and kidneys of animals trapped in lightly and moderately polluted sites and decreased for animals trapped in highly polluted sites. The SLI was negatively influenced by Pb concentrations in the liver. Finally, the SKI was positively linked to Cd concentrations in the kidneys. An increase of the relative liver and/or kidney mass has been found in individuals from different species that live in contaminated sites compared to individuals from control sites [6], [7], [35], [36], [38]. The higher relative masses of the liver and kidneys were interpreted as structural alterations and oedema formations caused by exposure to elements or compounds at toxic levels [6], [35], [38]. However, some authors failed to detect an adverse effect on relative organ mass [39]. We even found one study showing a reduced relative kidney mass in individuals from polluted sites compared to animals from reference populations [8]. Those findings highlight the difficulty of interpreting somatic indices as markers of TM toxicity.

Apart from the strong site-specific variability of indices, one of the most relevant study findings is that both body condition and somatic indices were related to TM concentration in organs and the degree of environmental contamination. The negative relationship between internal TM concentrations and indices in highly polluted sites has not been studied, but one could hypothesise that this negative effect is due to the reduction of food quality or food availability in the most contaminated sites.

### Conclusion

Most previous studies used body condition and somatic indices to discriminate populations inhabiting reference and polluted sites. In such a framework, most studies found lower body condition indices in individuals from polluted sites, suggesting a globally lower nutritional status in populations from contaminated areas. Similarly, liver and kidney indices usually showed higher relative organ masses in individuals from contaminated areas compared to those of reference animals, suggesting histopathological alterations or oedema. However, no direct evidence related those differences to pollution rather than other disturbances, such as food availability, food quality, or other variables that are rarely (if ever) studied in ecotoxicological studies. The present work used a large sample size of 560 individuals from 30 sites around a former Pb and Zn smelter, and we found slight yet significant relationships between body condition and liver indices, with interactions between Cd in the environment and Cd in the liver and kidneys. Relationships were significant between the liver index and Pb in the liver and between the kidney index and Cd in kidneys. Landscape, a variable used as an indirect indicator of habitat suitability, did not influence the indices, but the variability of indices between individuals sampled in different sites of capture was important. Studies based on the comparison of one reference and one contaminated site may be biased, with site-specific effects that may lead to misinterpretation of the results. Our work used indices calculated with a method that should consider allometry (due to gender dimorphism and growth) better than more classical methods (residuals form an ordinary least square regression), yet our indices were still influenced by the age of individuals (and gender for the body condition). Literature data show that indices may be influenced by season, food quality, or reproductive status. Based on the present results and literature data, we argue that indices are highly influenced by many biological and environmental parameters, and caution should be observed during index interpretation as an indicator of exposure to toxic elements or compounds. Even when used as part of a battery of parameters (histology, haematology, or biochemical biomarkers), the causal relationship between those indices and exposure to pollutants is rarely straightforward. For instance, it is well known that many histological alterations or haematological parameter variations may be due to infectious diseases, parasitism, or low quality and/or quantity of food [13]. Causal relationships between classical biomarkers used in wildlife ecotoxicology and various natural and anthropogenic stressors with which animals have to cope are greatly needed further research topics. Such research is the purpose of *stress ecology*, an area of research for which several authors have advocated [40]–[42].

## Acknowledgments

The authors gratefully thank Nadia Crini, Brice Mourier and Dominique Rieffel for their technical help and Cécile Grand from the *Agence De l’Environnement et de la Maîtrise de l’Energie* (ADEME) for fruitful scientific discussions. Jordi Peig is gratefully acknowledged for his help with calculation of the scaled mass index.
